# Central Nervous System Toxoplasmosis and Cytomegalovirus Colitis in an Asymptomatic HIV Positive Patient

**DOI:** 10.7759/cureus.17683

**Published:** 2021-09-03

**Authors:** Bernard O Emuze, Molly S Jain, Enkhmaa Luvsannyam, Paro Bhaya, Carlos Vaquero

**Affiliations:** 1 Emergency Medicine, St. James School of Medicine, Fort Worth, USA; 2 Medicine, St. James School of Medicine, Park Ridge, USA; 3 Surgery, Avalon University School of Medicine, Willemstad, CUW; 4 Neurology, American Doctor of Medicine (USMD) Program, Tbilisi State Medical University, Tbilisi, GEO; 5 Internal Medicine, MacNeal Hospital, Chicago, USA

**Keywords:** opportunistic infections, toxoplasmosis, cmv colitis, cd4 count, antiretroviral therapy, hiv

## Abstract

Human immunodeficiency virus (HIV) associated opportunistic infections are complications of patients with advanced HIV infection who are unaware of their disease or non-compliant with antiretroviral therapy. HIV patients with low CD4 count, generally less than 200 cells/μL, are at risk of developing opportunistic infections. We report a case of a 53-year-old male diagnosed with opportunistic infections, *Toxoplasma gondii *and cytomegalovirus (CMV). His initial presentation was central nervous system Toxoplasmosis and he later developed CMV colitis. Both are consequences of his undiagnosed advanced HIV status. The patient was promptly treated with appropriate medications for both conditions. The patient’s HIV status is well controlled with highly active antiretroviral therapy (HAART) and his CD4 count is improving. It further exhibits the significance of adequate screening protocols and the importance of early treatment for HIV patients.

## Introduction

Human immunodeficiency virus (HIV), the virus that causes acquired immunodeficiency syndrome (AIDS) has been an important global pandemic with mortality estimates affecting 39 million people worldwide [1]. HIV is transmitted via various modes such as blood transfusions, sexual, intravenous needle sharing, and from infected transplacental [1]. The pathophysiology of the retrovirus revolves around attacking the primary CD4 T lymphocytes causing severe immune dysregulation [2]. When the CD4 count drops to 200 cells/μL or less, the patient is at risk of AIDS-defining illness [2]. Some of the well-known opportunistic infections comprise viral, bacterial, fungal, and protozoal infections such as *Toxoplasma gondii*, *Pneumocystis jirovecii*, *Cryptococcus neoformans*, *Mycobacterium avium*, *Mycobacterium tuberculosis*, cytomegalovirus (CMV), herpes simplex viruses, and histoplasma capsulatum [2]. The diagnosis of HIV infection includes both screening and confirmatory testing. Measurement of CD4 counts, viral loads, and specific antibodies and antigens is the key to the management of the disease [1,2]. Antiretroviral therapy (ART) is crucial in the management of these patients. It suppresses the viral load and increases the CD4 T cell count [1,2].

When the patients' CD4 T count drops less than 100cells/μL, they are at the risk of acquiring toxoplasmosis, a protozoal infection [3]. It can present various forms such as encephalitis, pneumonitis, chorioretinitis, or disseminated infection in HIV-infected patients [3]. Cerebral toxoplasmosis can present with focal neurological symptoms such as hemiparesis, altered sensorium, ambulatory gait, speech abnormalities, severe headache, and seizures [4]. Cerebral toxoplasmosis usually presents as hypodense lesions with ring enhancement and perilesional edema on radiological imaging [3,4]. Treatment includes various drug regimens with the first line being a combination of sulfadiazine and pyrimethamine with the rescue of leucovorin for preventing folate deficiency [3,4]. Avoidance of eating raw or undercooked meat, handling cat litter, and proper handwashing techniques are major ways of prevention of toxoplasmosis infection [4]. 

The other viral infection common in HIV patients, especially when their CD4 count drops between 50 cells/μL to 100 cells/μL is CMV [5]. Though CMV commonly manifests as retinitis, it can also affect the pulmonary system causing pneumonitis, or the gastrointestinal system causing either esophagitis or colitis [5]. CMV colitis presents with non-specific symptoms as abdominal pain, hematochezia, fever, weight loss, and diarrhea [6]. Diagnosis is based on imaging techniques such as a CT scan of the abdomen; however, invasive techniques such as endoscopy and biopsy could be used if imaging studies are non-confirmatory [6]. Furthermore, CMV serology could be used to check the viral load and helps if treatment modification is needed [6]. Treatment drug regimen includes ganciclovir, valganciclovir and foscarnet [6]. 

We describe a case of a 53-year-old homosexual HIV-positive male who was diagnosed with central nervous system (CNS) toxoplasmosis and subsequently CMV colitis resulting in serious manifestations and clinical implications.

## Case presentation

We present a case of a 53-year-old male with a past medical history of anxiety disorder, depression, and cerebrovascular accident who presented to the emergency department (ED) complaining of fever, behavior changes, generalized weakness, vision issues, and headaches. The patient does not smoke, drink alcohol, or use any illicit drugs. He is allergic to sulfa antibiotics. In the ED, the patient further complained of worsening vision changes with more blind spots. On physical examination, his temperature was 100.5 F, head tender to touch, and pupils were equal, reactive and round to light and accommodation. The patient was slightly anemic (Hgb 12.2), but no other significant abnormalities were found on the lab test. Furthermore, the patient’s speech and thought processing were found slower than usual. He had no slurring of speech or facial drooping. The patient's symptoms have resolved on their own and he was stabilized until he returned to the ED with aggravation of symptoms three weeks later.

**Figure 1:**
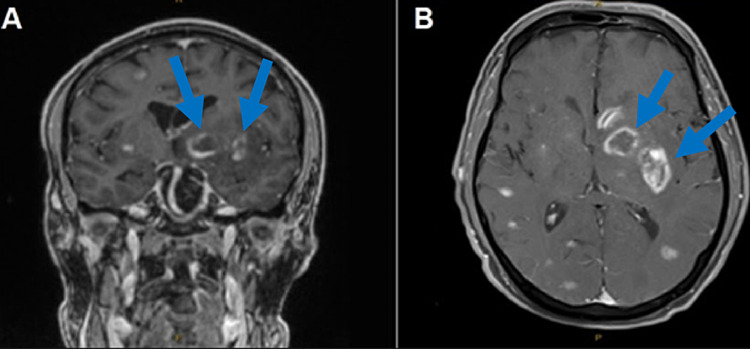
MRI of the brain demonstrating multiple ring-enhancing lesions (blue arrows) in coronal (A) and axial (B) sections.

The patient was admitted to the hospital due to persistent diffuse throbbing headache, weakness, ataxia, intermittent visual hallucinations, memory loss, and unintentional 10-lb weight loss. On a physical exam, the patient had a positive Romberg sign, hyper reflexia, increased muscle tone, left hemiparesis, and a flat affect. No generalized lymphadenopathy was found on the exam. No retinopathy was seen on fundoscopic examination. TSH, free T4, and vitamin B12 levels were within a normal range ruling out the thyroid issues and subacute combined degeneration of the spinal cord. Magnetic resonance imaging (MRI) of the brain T2-weighted image showed multifocal ring-enhancing lesions and vasogenic edema (Figure 1).

Additionally, an HIV antigen and antibody blood test was conducted and came back positive. His CD4 T lymphocyte count was less than 50 cells/μL. The patient was unaware of his HIV status and has been asymptomatic for years. Upon further inquiry, the patient mentioned that he is homosexual and his HIV testing was negative five years ago. The patient is sexually active with one partner and denied similar illness in his partner in the past. He was educated on the importance of informing his partner and have him tested as well. A confirmatory test was positive with the presence of HIV-1 antibodies. The patient was started on highly active antiretroviral therapy (HAART), bictegravir-emtricitab-tenofovir. 

**Figure 2:**
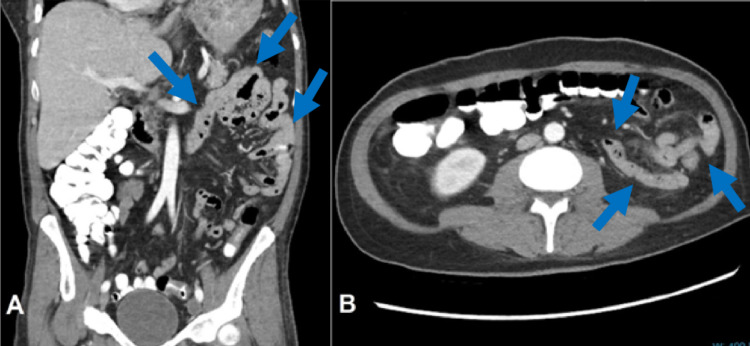
CT of the abdomen and pelvis with contrast revealing diffuse bowel thickening of the descending and sigmoid colon as well as the rectum in coronal (A) and axial (B) sections (blue arrows).

Multiple ring-enhancing lesions in the brain in the setting of HIV were concerning for toxoplasmosis or lymphoma. Hence, a biopsy of the multifocal ring-enhancing lesions was performed which confirmed CNS toxoplasmosis. The serological testing also detected toxoplasma IgG antibodies. The patient was placed on atovaquone 1,500 mg, leucovorin 20 mg, and pyrimethamine 75 mg daily for prompt treatment. The patient reported no allergic symptoms to pyrimethamine. During his hospital stay, the patient also developed generalized abdominal pain and diarrhea. A CT of the abdomen and pelvis with contrast revealed diffuse wall thickening of the descending and sigmoid colon as well as the rectum consistent with colitis (Figure 2).

Due to the patient’s immunocompromised state, serology testing for CMV antibodies confirmed elevated levels, and the CMV viral load was significantly increased. The patient was started on valganciclovir 900 mg. Further testing was conducted for the presence of other opportunistic organisms which was negative. The patient is tolerating the HAART and his CD4 count is checked regularly. The HIV viral load was 173,489 copies/mL before HAART and it dropped to <40 copies/mL after the HAART. Few weeks after the initiation of HAART, his CD4 T lymphocyte count has increased up to 80 cells/μL. The patient was discharged once he was stabilized. 

During one-week outpatient follow-up, the patient reported feeling intermittent dizziness; however, his headaches, vision issues, and hallucinations were diminished. Three months after the initial diagnosis the patient has started doing physical therapy sessions and his ataxia is slowly improving. The patient was educated on the importance of medication adherence and regular clinical assessment.

## Discussion

Toxoplasmosis is a serious disease with major morbidity and mortality in the US and globally. The parasite, *T. gondii*, can be transmitted from ingesting raw meat containing the cysts or water infested with the oocysts from feline feces [7]. The vertical transmission during pregnancy leads to congenital toxoplasmosis and the rarest transmission can occur through transplanted organs as well [7]. Worldwide more than six billion people have been affected with *T. gondii* [7]. The prevalence of toxoplasmosis has been decreasing in the US; however, it is still the second most common cause of death from foodborne diseases [7]. There are one million new infections reported with 750 deaths annually [7]. As one of the opportunistic infections, *T. gondii* can occur more frequently and severely in immunocompromised patients. The disease presentation varies in each patient and can present with serious eye and CNS infections in immunocompromised patients [7]. Although vision loss is uncommon, there are approximately 20,000 cases of retinal infection with *T. gondii* reported [7].

The incidence of opportunistic infections is a major problem in people who are unaware of their HIV status or those noncompliant with the treatment [8]. The HAART increases the CD4 lymphocyte count and reduces the viral replication improving the host defense mechanism and the chance of survival [8]. Despite the ongoing development of HAART, the prevalence of opportunistic infections is still high in patients who are compliant with their medications and toxoplasmic encephalitis remains to be the major cause of mortality in AIDS patients [7,8].

HIV infection weakens the immune system increasing the risk of life-threatening opportunistic infections. The opportunistic infections also increase HIV progression and transmission by boosting the viral load [9]. The type of opportunistic infections depends on the level of patient immunity. Milder infections such as skin infection by herpes zoster occur in an early stage, however, more serious infections such as CNS toxoplasmosis or cryptococcal meningitis can occur with severe immune suppression in later stages [9]. CNS infection can present with nonspecific symptoms such as headache, lethargy, dementia, hallucinations, and seizures making the clinical diagnosis difficult [7]. Our patient initially presented with headache, visual disturbances, ataxia, and weakness, which was attributed to an embolic event. Three weeks later, when the patient returned to the hospital with severe CNS symptoms such as dementia, visual hallucinations, and hemiparesis, he was finally diagnosed HIV positive and CNS toxoplasmosis.

CMV infection is common and transmitted through close contact with bodily fluids including saliva, mucus, urine, blood, and tears [10]. The host immune response plays a role in controlling CMV infection. Primary CMV infection is usually asymptomatic or mild in immunocompetent hosts and it stays in a life-long latent phase [10]. CMV reactivation from the latent phase due to immunosuppression can cause serious disease in immunocompromised people [10]. During the hospital stay, our patient developed abdominal pain and diarrhea which was confirmed to be CMV colitis. His immunocompromised state led to the reactivation of the virus in the latent stage causing his symptoms.

The highlight of this case focuses on the importance of prompt diagnosis and treatment of HIV/AIDS. Immunosuppression can cause life-threatening opportunistic infections as seen in our patients. Due to nonspecific symptoms of some infections, HIV can be misdiagnosed causing delayed treatment and a higher risk of complications. Toxoplasmosis has not only high prevalence in regions such as South America, but the disease can also present in any patient with immunosuppression. Primary care clinicians should recommend HIV screening to all adolescents and adults aged 15 to 65 years. Younger adolescents and older adults who are at increased risk should also be screened more often. Patients should be educated on the importance of all sexually transmitted disease testing including HIV and prevention of its complications. An appropriate screening protocol will prevent the disease or can help diagnose and initiate treatments of a specific disease in a timely manner. This will keep the CD4 T lymphocyte count and the viral load in control, therefore, increasing the patient’s chance of survival.

## Conclusions

Once diagnosed, the management of HIV is lifelong. Hence, the patient's education on disease transmission, benefits of ART, and awareness of opportunistic infections are important. CNS toxoplasmosis, if not diagnosed and treated on time, can cause detrimental complications such as seizures, dementia, and even death. It must be considered a differential in immunocompromised patients with neurological symptoms with unknown etiology, especially in the setting of advanced HIV infection. This case has established the importance of adequate HIV screening to avoid disease progression and life-threatening opportunistic infections. Prompt diagnosis and treatment of early HIV infection decrease patient mortality considerably. Finally, when individual patient needs are addressed, a multidisciplinary approach will provide a stronger outcome and quality of life.
